# Autophagy plays an essential role in ultraviolet radiation-driven skin photoaging

**DOI:** 10.3389/fphar.2022.864331

**Published:** 2022-10-06

**Authors:** Jingwen Ma, Yan Teng, Youming Huang, Xiaohua Tao, Yibin Fan

**Affiliations:** ^1^ Medical Cosmetic Center, Shanghai Skin Disease Hospital, Skin Disease Hospital of Tongji University, Shanghai, China; ^2^ Health Management Center, Department of Dermatology, Zhejiang Provincial People's Hospital, Affiliated People's Hospital, Hangzhou Medical College, Hangzhou, China

**Keywords:** autophagy, photoaging, ultraviolet, UV, skin aging

## Abstract

Photoaging is characterized by a chronic inflammatory response to UV light. One of the most prominent features of cutaneous photoaging is wrinkling, which is due primarily to a loss of collagen fibers and deposits of abnormal degenerative elastotic material within the dermis (actinic elastosis). These changes are thought to be mediated by inflammation, with subsequent upregulation of extracellular matrix-degrading proteases and down-regulation of collagen synthesis. Autophagy is a vital homeostatic cellular process of either clearing surplus or damaged cell components notably lipids and proteins or recycling the content of the cells’ cytoplasm to promote cell survival and adaptive responses during starvation and other oxidative and/or genotoxic stress conditions. Autophagy may also become a means of supplying nutrients to maintain a high cellular proliferation rate when needed. It has been suggested that loss of autophagy leads to both photodamage and the initiation of photoaging in UV exposed skin. Moreover, UV radiation of sunlight is capable of regulating a number of autophagy-linked genes. This review will focus on the protective effect of autophagy in the skin cells damaged by UV radiation. We hope to draw attention to the significance of autophagy regulation in the prevention and treatment of skin photoaging.

## Introduction

Skin photoaging describes changes in clinical, histological, and functional characteristics of elderly skin that can be observed in areas that are exposed to sunlight (Gilchrest, 2013). The clinical features of skin photoaging are fine lines, wrinkles, discoloration, telangiectasias, and roughened appearance. These changes are related to the pathophysiology of various cells and tissues in both the epidermis and dermis. The leathery texture of skin chronically exposed to sunlight may be due to abnormal proliferation and the morphologic heterogeneity of keratinocytes in the basal cell layer. The discoloration of spots is mainly due to irregular melanosomes after solar irradiation. The most obvious manifestation of photoaging are wrinkles, which are mainly due to the loss of collagen fibers and the deposition of abnormally degraded elastic substances in the dermis (Han et al., 2014). Solar energy is mainly composed of ultraviolet (UV) light, visible light, and infrared rays. Despite recent studies suggesting that visible light, especially blue light, plays a role in photoaging (Pourang et al., 2021), UV radiation is thought to be the main cause of skin photoaging (Le Digabel et al., 2018).

Several studies have reported multiple pathways resulting in photoaging at the molecular level. DNA damage, telomere shortening, and matrix metalloproteinase (MMP) degradation are the main mechanisms of skin photoaging (Bosch et al., 2015), and p21, p53, p62, Lamin B1, TGF-β, HSP 27, and Lamp-1 are involved in the processes of photoaging (Endo et al., 2020; Bai et al., 2021). Autophagy is a fundamental process of cell metabolism that degrades proteins and organelles in cells and reuses these components in the most efficient way to maintain cell survival and tissue stability (CB KDJCb, 2005). The loss of autophagy may lead to the initiation of photoaging in UV-exposed skin (Yang et al., 2016; Cavinato et al., 2017). In addition, UV light activates a series of autophagy-related genes to affect downstream signal transduction (Bianco and Schumacher, 2018; Gu et al., 2020; Chen et al., 2021). The UV radiation resistance-associated gene (UVRAG) is an autophagy-related protein (Zhao et al., 2012) that maintains the integrity of DNA by activating the nucleotide excision repair pathway (Pourang et al., 2021). However, the mechanisms of autophagy in UV-induced skin photoaging remain unclear. This review summarizes the recent literature regarding UV-induced autophagy in skin and the role of autophagy in the skin cell response to UV exposure with the aim of highlighting the significance of autophagy regulation for the prevention and treatment of skin photoaging.

## Mechanisms of skin photoaging

Different layers of skin contain a variety of chromophores that can interact with UV radiation (Emri et al., 2018). These interactions include reflection, refraction, absorption, and transmission. The accumulation of energy absorption induced by UV light gradually promote skin aging. The UV components of sunlight, including UVA (320–400 nm) and UVB (280–320 nm) radiation, are associated with skin photoaging (Gilchrest, 2013). UVA radiation is divided into UVA-1 (340–400 nm) and UVA-2 (320–340 nm) (Mutzhas et al., 1981). UVA is more abundant (5.6% of the sunlight) than UVB (0.15% of the sunlight) (Battie and Verschoore, 2012), and 35–50% of UVA penetrates the dermis. Therefore, UVA plays a more important role in biological changes that induce skin photoaging (van den Akker et al., 2004). Within the dermis, UVA causes changes in structural and matrix proteins, resulting in the loss of collagen fibers and the rupture of elastic fibers (Nakyai et al., 2018). In contrast, UVB is highly biologically reactive, though most of it is absorbed or dissipated by the proteins and nucleic acids in the epidermis, resulting in the production of pyrimidine dimers and damage to telomerases (Yang et al., 2019). The combined effects of UVA and UVB lead to cell injury, inflammation, immunosuppression, extracellular matrix remodeling, and angiogenesis.

### Direct damage to DNA

DNA is the primary target of UV-induced cell damage. UV radiation between 245–290 nm is absorbed by DNA (Tornaletti and Pfeifer, 1996), inducing the formation of cyclobutene pyrimidine dimers and pyrimidine-(6–4)-pyrimidone photoproducts (Ewing et al., 2008; Bérubé et al., 2018). These DNA mutations may be associated with specific symptoms of photoaging, such as wrinkles, elastin breakage, and collagen damage (Bissett et al., 1989). Chronic low-dose UVB radiation-induced residual cyclobutane pyrimidine dimers (CPDs) remains, catalyzing sister chromatin exchange (Bérubé et al., 2018), suggesting that UVB irradiation may lead to DNA damage and epidermal cell apoptosis (Matsumura and Ananthaswamy, 2004).

UVA induces a cyclobutane pyrimidine dimer (CPD) other than CPDs which may also lead to DNA damage (Tewari et al., 2012). Chronic UVA-1 irradiation leads to DNA damage in human dermal fibroblasts (Montoni et al., 2019).

### Indirect damage through inflammation and immunosuppression

UV directly and indirectly affects the formation of pyrimidine dimers by DNA through reactive oxygen species (ROS) (Cadet et al., 2000), including superoxide anion, peroxide, and singlet oxygen (Sies and Jones, 2020). UVA and UVB radiation may induce the production of ROS in mammalian cells (He et al., 2005). ROS production is the most critical step in all molecular reactions of human skin when exposed to UV radiation (Fisher et al., 2002). The mechanism of receptor activation by UV irradiation is not well understood, but it has been suggested that ROS inhibit specific protein-tyrosine phosphatases, resulting in increased receptor activation (Gross et al., 1999).

UV radiation may mimic the actions of receptor ligands via the generation of ROS via its *in vitro* effects on the extracellular matrix (Heck et al., 2004). As important regulators of collagen metabolism, ROS contribute to tissue oxidation and degradation and interfere with signal transduction involved in gene expression (Wlaschek et al., 2001). It has been hypothesized that DNA damage and ROS production trigger an inflammatory response that changes the cell structure and function. Changes in intracellular homeostasis and the production of ROS activate intracellular multiprotein platforms (Harijith et al., 2014).

Almost all nucleated cells produce cytokines when stimulated by UV irradiation, including keratinocytes, melanocytes, dermal fibroblasts, sebocytes, endothelial cells, smooth muscle cells, mast cells, lymphocytes, and other inflammatory cells (Hart et al., 2000; Walterscheid and DXNghiemUllrich, 2002). TGF-β and platelet-derived growth factor are two major cytokines that modulate dermal alterations in skin exposed to UV radiation. TNF-α, IL-1, and IL-6 play important roles in maintaining the homeostasis of photodamaged cells (Zhang et al., 1998; Kondo, 2000).

UV exposure has also been associated with local and systemic immunosuppression (Beissert and Schwarz, 2003), which may be related to cutaneous tumor surveillance (Bosch et al., 2015). UV radiation inhibits immunity through complex pathways, initiated by chromophores in the skin and ending with the generation of specific subsets of T and B regulatory cells and the inhibition of effector and memory T cell activation (Norval and Woods, 2011). A reduction in contact hypersensitivity reactions (Noonan et al., 1981) and delayed-type hypersensitivity (Strickland et al., 1994) has been observed after UV radiation exposure. This immunosuppression is mediated in part by DNA damage and altered cytokine expression (Vink et al., 1997). UV-induced immunosuppression may serve to prevent the autoimmune response of inflammatory products caused by UV-mediated injuries. Receptors for epidermal growth factor, IL-1, and TNF-α are activated in keratinocytes and fibroblasts within 15 min of UV exposure (Wang et al., 2005). Inflammasomes nucleate around the cytoplasmic receptors in the nucleotide-binding domain and leucine-rich repeat families, regulating the secretion of caspase-1-dependant pro-inflammatory IL-1β and IL-18, altering the cell structure and function (Awad et al., 2018).

### Accumulation of MMPs and reduction of collagens

The collagen synthesis by fibroblasts decreases while the synthesis of MMPs increases due to direct UV damage and UV-induced inflammatory factors and cytokines. These changes result in collagen reduction and elastic fiber fractures, leading to wrinkles, fine lines, and reduced skin elasticity.

MMPs are responsible for degrading the extracellular matrix (Choi et al., 2020) and are located in epidermal keratinocytes and dermal fibroblasts (Fisher et al., 1997). MMPs include MMP-1 (a collagenase), MMP-3 (stromelysin), and MMP-9 (92-kd gelatinase) (Brinckerhoff and Benbow, 1997). There is a dose-response relationship between UV exposure and MMP induction (Bulteau et al., 2007). UV radiation may activate the ROS/MAPK/AP-1 pathway in human keratinocytes and dermal fibroblasts (Kim et al., 2018). ROS stimulate cell surface receptors, resulting in membrane lipid damage, leading to the release of ceramides and the activation of nuclear transcription factor activator protein 1 (AP-1) (Yaar, 2007), which controls the expression of MMPs (Brinckerhoff and Benbow, 1997).

Collagen production is reduced in photoaged skin, leading to wrinkles (Talwar et al., 1995). After UV irradiation, procollagen significantly decreases, disappearing 24 h after irradiation (Fisher et al., 2000). AP-1 and TGF-β are involved in the UV-mediated down-regulation of collagen synthesis (Briganti and Picardo, 2003). AP-1 consists of two subunits: constitutively expressed c-Fos and the UV-inducible c-Jun (Fisher et al., 1996). Overexpression of the c-Jun component of AP-1 in cultured fibroblasts reduces the expression of type I collagen (Fisher et al., 2000). TGF-β is an important promoter of collagen synthesis (Chung et al., 1996). TGF-β and its receptor are decreased in the epidermis and dermis after UV irradiation (Quan et al., 2002). The impaired diffusion and adhesion of fibroblasts during collagen degradation may inhibit collagen synthesis. This results in a cycle of reduced production of new collagen due to poor adhesion between fibroblasts and the damaged collagen, resulting in a gradual increase in photodamage (Varani et al., 2004). Long-term exposure to UV radiation results in the gradual accumulation of MMPs and the reduction of collagen, which are considered to be the consequences of photoaging (Kang and Fisher, 2001).

## Autophagy protects cells from UV damage through different pathways

### Autophagy

Autophagy is an intracellular homeostatic process for the turnover of cellular organelles and proteins that involves encapsulation, the formation of autophagosomes, fusion with lysosomes to form autolysosomes, and the degradation of the encapsulated contents (Bai et al., 2021). Autophagy includes three main classical pathways: macro autophagy, micro autophagy, and chaperone-mediated autophagy (Nie et al., 2021). The roles of autophagy in cardiovascular diseases, kidney diseases, infections, and autoimmune diseases are well-known (Bravo-San Pedro et al., 2017; Rajendran et al., 2019; Tang et al., 2020). The use of autophagy as a therapeutic target for these diseases may be beneficial (Nys et al., 2013; Zhong et al., 2016).

### Autophagy process

Autophagy is divided into four stages: initiation, extension, maturity, and termination. During initiation, a precursor structure (phagophore) with a double membrane is formed in the cell. During extension, the double membrane extends to envelop the substrate, forming an autophagosome. During maturity, the autophagosome is transported to a lysosome and fused to form an autolysosome (Dikic and Elazar, 2018). During termination, the substances encapsulated by the autophagosomes are degraded (Bai et al., 2021). The biomolecules and energy produced by this process help maintain normal metabolic processes, as autophagy products circulate in the cytoplasm, helping to restore important cellular processes after exposure to stressors (Pankiv et al., 2007).

### Autophagy Maintains Intracellular Homeostasis by removing Damaged DNA and Organelles

Autophagy is crucial for proper nucleotide excision repair in mammalian cells (Qiang et al., 2016). UVB radiation directly induces autophagy by activating AMP-activated protein kinase (AMPK) (Cao et al., 2008) which is sensed via an increase in the AMP/ATP ratio (Shin et al., 2016). UVB induces the phosphorylation of AMPK and increases the expression of downstream target genes. AMPK activation by UVB increases the autophagic flux (Sample and He, 2017).

Mechanistic target of rapamycin (mTOR) is a negative regulator of autophagy that controls cell growth and metabolism by integrating nutritional and stress signals. When stimulated by UV radiation, PI3K/Akt activation is induced by UVB radiation, which activates downstream mTOR to inhibit autophagy (Wei et al., 2012; Zhang et al., 2018). In contrast, the inhibition of mTOR promotes autophagy (Kim and Guan, 2015).

Autophagy repairs broken DNA duplexes via homologous recombination and non-homologous terminal junctions (LRGomesMenck and Leandro, 2017). Autophagy is also associated with nucleotide excision repair, base excision repair, and mismatch repair (SenGupta et al., 2013). Therefore, autophagy helps remove small base alterations in the DNA structure, correct errors in replication, and maintains intracellular homeostasis.

### Autophagy helps photoaged cells suppress inflammation

Autophagy helps maintain the balance and function of various immune cells, including neutrophils, B lymphocytes, T lymphocytes, and dendritic cells (Levine et al., 2011). The activation of autophagy due to oxidative stress has been well demonstrated in several biological models (Brinckerhoff and Benbow, 1997). Oxidative stress induced by the formation of ROS also induces autophagy (Filomeni et al., 2015). Autophagy protects against oxidative stress by clearing the damaged proteins, lipids, and DNA and restoring metabolic homeostasis (Wen et al., 2013). Autophagy inhibits inflammasomes and NLRP3 inflammasome activation, reducing the production of IL-1β (Biasizzo and Kopitar-Jerala, 2020). As a result, autophagy may alleviate damage induced by UV light via the inhibition of the inflammatory response and downregulation of the production of inflammatory factors.

### Autophagy helps remove excess MMP in photodamaged skin cells

**FIGURE 1:**
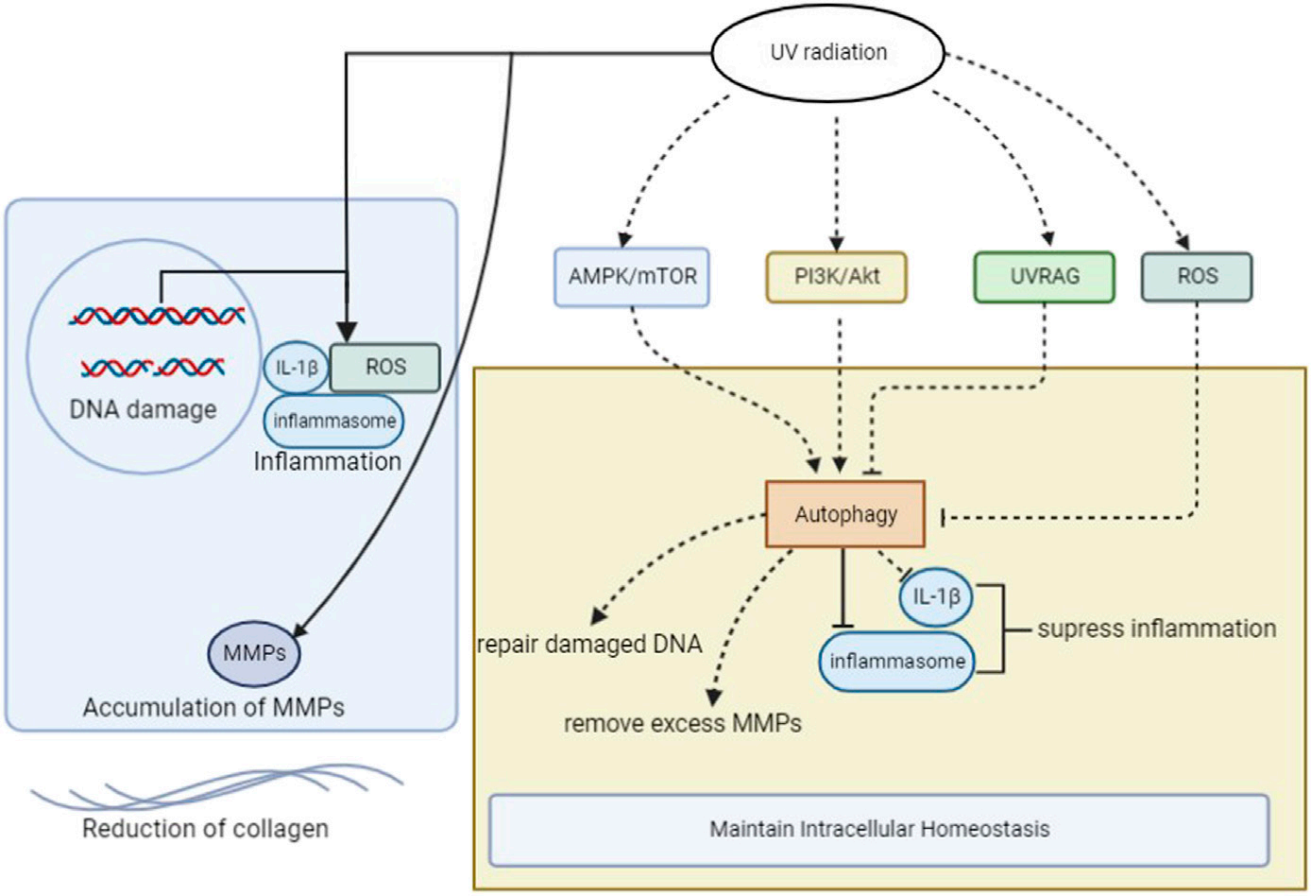
The mechanism of photoaging and the role of autophagy after UV radiation in photodamaged skin cells. ROS, reactive oxygen species; UV, ultraviolet; IL-1β, Interlukin-1β; UVRAG, UV Radiation Resistance Associated Genes; MMPs, matrix metalloproteinases.

The inhibition of autophagy downregulates the expression of TGF-β1, collagen I, and collagen III and increases the expression of MMP-2 and MMP-13 (Li et al., 2018a). Metformin induces autophagy via the AMPK/mTOR pathway, downregulating the expression of MMPs (Li et al., 2017). Therefore, autophagy may help remove excess MMPs and reduce collagen degradation in photodamaged cells (Figure 1). However, few studies have evaluated the relationship between autophagy and collagen, and more studies are needed.

## The role of autophagy in skin photoaging

**FIGURE 2:**
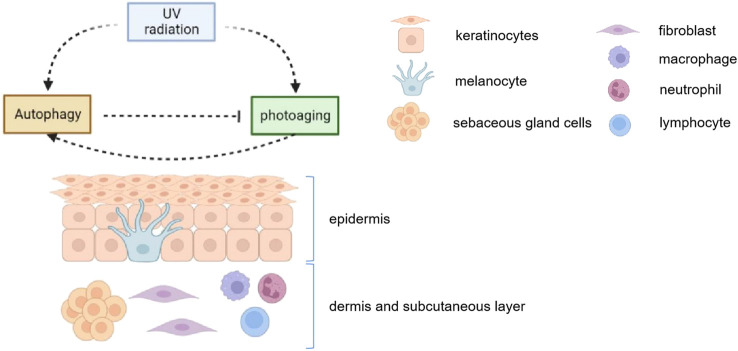
UV radiation activates autophagy in epidermal keratinocytes, melanocytes, dermal fibroblasts and sebaceous gland cells. Meanwhile, the induction of autophagy helps maintain the homeostasis in those cells. Autophagy 1) induces melanosome degradation *in vitro*, alleviates melanocyte inflammation and may protect against UV-induced pigmentation; 2) inhibits inflammatory response, prevents peroxidation in keratinocyets; 3) maintans the normal activity in fibroblasts after UV radiation; 4) regulates in lipids secretion and proportion in sebaceous glands. Therefore, autophagy plays an important role in maintaining the balance of skin cell senescense and cell stasis.

UV radiation activates autophagy in epidermal keratinocytes, melanocytes, dermal fibroblasts and sebaceous gland cells, leading to a series of downstream changes. And autophagy plays an important role in maintaining the balance of skin cell senescense and cell stasis (Figure 2).

### UV induces autophagy in keratinocytes

Keratinocytes are the dominant components of the epidermis, and their differentiation maintains the function of the skin. Autophagy is constitutionally active in the epidermal granular layer (Qiang et al., 2021). Impaired autophagy contributes to the pathogenesis of psoriasis, a disease characterized by keratinocyte keratosis (Boehncke and Schön, 1997). In patients with psoriasis, the skin lesions correlate with parakeratosis and the expression of autophagy-related proteins is not regulated (Akinduro et al., 2016). Knockdown of ATG5, one of the essential inducers of autophagy, inhibits keratinocyte proliferation and differentiation (Xie et al., 2016a), highlighting the important role of autophagy in keratinocyte differentiation.

UV-oxidized lipids are signaling mediators that convey cellular responses to oxidant stress in keratinocytes (Gruber et al., 2010). UVA radiation and UVA-oxidized phospholipids ignite autophagy in epidermal keratinocytes, which is considered critical for the environmental UV-stress degradation of proteins and lipids. Both UVA and UVA-oxidized phospholipids induce autophagy in epidermal keratinocytes. Without autophagy, the degradation of UV-modified molecules, such as oxidized lipids and aggregated proteins that contribute to tissue damage, is impaired. During homeostasis, autophagy prevents the accumulation of oxidized phospholipids and the overexpression of Nrf2 target genes in keratinocytes (Endo et al., 2020).

ROS play an important role in the process of photoaging. Mitochondrial ROS produced during oxidative phosphorylation in suprabasal keratinocytes trigger autophagy and lysosome-mediated degradation, contributing to epidermal differentiation (Monteleon et al., 2018). FGF21 plays a critical role in enhancing stress resistance, such as UV-irradiation tolerance and thermotolerance (Song et al., 2020). FGF21 promotes the migration and differentiation of epidermal cells during wound healing via SIRT1-dependent autophagy (Gu et al., 2020). FGF7 also induces autophagy, promoting keratinocyte differentiation (Belleudi et al., 2014). Various pathophysiological processes, including wound repair and inflammation, are upregulated in psoriatic lesions, suppressing inflammation in keratinocytes via the promotion of autophagy through the Wnt/β-catenin signal pathway. UVB radiation downregulates ULK1 and ATG7 expression and impairs the autophagy response in human keratinocytes (Chen et al., 2018).

### UV induces autophagy in human dermal fibroblasts

Autophagy promotes TGF-β-induced fibroblast activation (Zhang et al., 2020). Autophagy has been observed in photoaged fibroblasts (Zhang et al., 2016). *In vitro*, autophagy-related protein levels are significantly higher in UVA-exposed fibroblasts than in non-photoaged control cells. The autophagic flux is induced in photoaged fibroblasts treated with rapamycin, suggesting that UVA-induced photoaging may inhibit autophagy at the degradation stage (Cavinato and Jansen-Durr, 2017). Interestingly, short-term UVA irradiation induces autophagy, and long-term UVA irradiation inhibits autophagy both *in vitro* and *in vivo* (Xie et al., 2021).

Autophagy is required for the establishment of the senescent phenotype in UVB-treated fibroblasts, and the inhibition of autophagy is sufficient to change the cell’s fate from senescence to death by apoptosis (Yang et al., 2016). Autophagy promotes dermal fibroblast differentiation and collagen production by regulating reticulum stress and autophagy-dependent secretion (Zhou et al., 2021). Increased ROS production and the inhibition of proteasomes followed by autophagy activation are early events in the process of UVB-induced senescence of human dermal fibroblasts.

In addition, different doses of UVB irradiation have different effects on the autophagy function of human skin fibroblasts. *In vitro*, low doses of UVB can directly upregulate the autophagy function of cells, while high doses of UVB can seriously damage cells, leading to significant changes in cell morphology and the downregulation of autophagy (Moon et al., 2019). Acute light injury caused by UVB results in increased protein and organelle damage, and autophagy maintains the stability of the intracellular environment (Wang et al., 2008; Moon et al., 2019). Long-term UVB exposure downregulates autophagy in human dermal fibroblasts by inhibiting UVRAG or activating the PI3K/Akt pathway to act on the mTOR receptor, leading to the downregulation of autophagy.

Due to the serious decline of physiological function when UV damage accumulates, photoaged skin fibroblasts do not respond effectively to external stimuli and maintain the stability of the internal environment. Therefore, autophagy is downregulated in photoaged fibroblasts, aggravating the decline of its physiological function, forming a vicious circle (Umar et al., 2021).

### Autophagy and photoaging in melanocytes

Autophagy and the regulators of autophagy play a wide variety of roles in melanocyte biology (Zhang et al., 2015; Qiao et al., 2016). The activation of autophagy induces melanogenesis and regulates melanosome biogenesis in melanocytes (Jeong et al., 2020). Autophagy induction regulates the physiologic skin color via melanosome degradation, although the downstream effectors are unclear (Murase et al., 2013). Increased autophagic flux may lead to melanosome degradation *in vitro* (Kim et al., 2020). Autophagy deficits may lead to melanocyte hyperfunction in several pigment disorders associated with photoaging, including melasma (Espósito et al., 2021).

UVRAG, which was originally identified as a BECN1-binding autophagy protein, has a specialized function in melanosome biogenesis beyond autophagy through its interaction with the biogenesis of lysosome-related organelles complex 1. This melanogenic function of UVRAG is controlled by the melanocyte-specific transcription factor MITF as a downstream effector of the α-melanocyte-stimulating hormone-cAMP that signals as part of the suntan response (Li et al., 2019).

Melanocytes are particularly vulnerable to oxidative stress due to their pro-oxidant state, that is, generated during melanin synthesis and genetic antioxidant defects (Seo and Fisher, 2010). UVB exposure may increase ROS production, increasing autophagy in normal melanocytes to protect cells from oxidative damage (Murase et al., 2013; Xie et al., 2016b). Low intensity UVA may increase intracellular ROS production and decrease mitochondrial membrane potential, inducing autophagy (Yumnam et al., 2021). If autophagy is dysregulated by the impairment of the Nrf2-p62 pathway, the sensitivity of melanocytes to oxidative stress due to UV exposure is increased (He et al., 2017).

### The role of autophagy in UV-exposed sebaceous glands

UV radiation may increase the production of singlet oxygen and free radicals on the skin surface, which are important oxidants for skin lipids (Akitomo et al., 2003). Oxidation of skin surface lipids may play an important role in sunburn, wrinkle formation, hyperpigmentation, freckles, acne, atopic dermatitis, and cancer (Bickers and Athar, 2006). Autophagy contributes to sebaceous gland function and to the control of sebum composition (Koenig et al., 2020; Seo et al., 2020). Autophagy markers are strongly expressed in maturing sebaceous gland cells in healthy skin (Seo et al., 2020). The inactivation of autophagy leads to changes in sebaceous gland morphology and function (Rossiter et al., 2018). The free fatty acid to cholesterol ratio decreases and the ratio of fatty acid to methyl ester increases in mutant mice when the autophagy-related gene Atg4 is knocked out (Rossiter et al., 2018), suggesting that autophagy contributes to sebaceous gland function and the control of sebum composition. Pharmacological inhibition of autophagy leads to increased sebaceous lipid accumulation (Fischer et al., 2017).

The activation of AMPK inhibits the lipid secretion of primary human sebocytes (Yang et al., 2021). The activation of the mTOR signaling pathway stimulates sebaceous glands to accelerate sebum secretion (Tuo et al., 2017). In sebocytes, the activation of the PI3K/Akt and mTOR pathways that induce high protein and lipid synthesis increases cell growth, proliferation, and inflammation (Mastrofrancesco et al., 2017). UV radiation may affect autophagy by activating the PI3K/Akt/mTOR pathway, thus affecting sebaceous gland secretion. However, few studies have reported the direct relationship between autophagy and UV exposure in sebaceous glands; more studies are necessary.

### The protective effect of autophagy is limited

It is worth noting that the protective effect of autophagy is limited. Repetitive UVA irradiation interferes with the autophagy process. Short-term UVA irradiation induces autophagy via the stimulation of autophagosome formation, which occurs during early autophagy, and increases the number of lysosomes (Endo et al., 2020). However, chronic UVA exposure causes lysosomal dysfunction in skin fibroblasts (Lamore, 2013; Huang et al., 2019). Excessive UVB irradiation also leads to decreased autophagy functions in fibroblasts *in vitro* (Ali and Sultana, 2012), suggesting that autophagy homeostasis may be disrupted by sustained or excessive UV exposure.

## Autophagy inducers: An alternative option in preventing skin photoaging

Autophagy suppresses the activation of inflammasomes by regulating the mTOR and AMPK pathways, inhibiting the inflammatory responses induced during photosenescence. Autophagy also helps regulate the inflammatory responses through the NF-kappa B pathway, which is beneficial to the repair of DNA-damage in photoaged cells. Autophagy modulates endothelial junctions to restrain neutrophil diapedesis during inflammation (Reglero-Real et al., 2021).

### Rapamycin

Clinical trials that explore the protective effects of several autophagy inducers on photoaging have been conducted. Rapamycin is the most commonly used autophagy inducer. Rapamycin binds to mTOR, inhibiting the mTOR signaling pathway and promoting autophagy (Benjamin et al., 2011). *In vivo*, rapamycin protects skin fibroblasts from UVA-induced photoaging via the inhibition of p53 and phosphorylated HSP27 (Bai et al., 2021). *In vitro*, rapamycin may also attenuate the radiation-induced release of ROS by reducing ROS accumulation in photoaged fibroblasts (Qin et al., 2018).

### Green tea

Some studies suggest that green tea (*Camellia sinensis*) supplementation protects against wrinkles by increasing collagen and elastin fiber levels and inhibiting the production of MMP-3 in the skin (Kowalska et al., 2021). Recent studies have reported that green tea can activate autophagy by increasing the activity of PI3 kinase and BECLIN-1 (Prasanth et al., 2019).

### Neuropeptide α-neoendorphin (NEP)

The opioid neuropeptide α-neoendorphin (NEP) is an endogenous decapeptide that activates the kappa opioid receptor and exhibits certain anti-aging and anti-wrinkling effects on skin cells. NEP activates cellular autophagy via the mTOR-Beclin-1-mediated signaling pathway in dermal fibroblasts. In murine models, NEP increases type I procollagen production and decreases MMP-1, MMP-2, and MMP-9 activities (Lim et al., 2020). Astragaloside exerts anti-photoaging effects in UVB-induced, premature senescence of rat dermal fibroblasts through enhanced autophagy (Wen et al., 2018).

### Aquatide and caffeine

Treatment with Aquatide directly activates SIRT1 and induces autophagy in cultured human dermal fibroblasts. Aquatide is a novel synthetic SIRT1 activator (heptasodium hexacarboxymethyl dipeptide-12) and modulates autophagy through SIRT1 activation, contributing to the suppression of skin aging caused by UV irradiation.

In addition, caffeine has been reported to protect skin from oxidative stress-induced senescence through activating the A2AR/SIRT3/AMPK-mediated autophagy (Li et al., 2018b).

## Conclusion and future directions

UV irradiation plays a major role in inducing photoaging by increasing the levels of oxidative lipids and metabolite aggregation. Autophagy plays an essential role in photodamaged skin. Autophagy is upregulated by UV radiation in several skin cells and removes damaged DNA and organelles to maintain the balance of the internal environment in photodamaged skin. In contrast, autophagy dysfunction is likely to promote UV-induced skin cell damage. Although great progress has been made in the fight against UV-induced photoaging, the role of autophagy is unclear. Autophagy inducers can be considered as a novel treatment for the prevention or reversal of photoaging.
